# Supplementary material for: Polycomb condensates can promote epigenetic marks but are not required for sustained chromatin compaction
Source: Nat Commun. 2021 Oct 7;12:5888. doi: 10.1038/s41467-021-26147-5 (PMC8497513; doi:10.1038/s41467-021-26147-5)
Supplement: Supplementary file 1 — Supplementary Information [file 41467_2021_26147_MOESM1_ESM.pdf]

SUPPLEMENTARY INFORMATION

Supplementary Figures S1-S4

Supplementary Table S1-S2

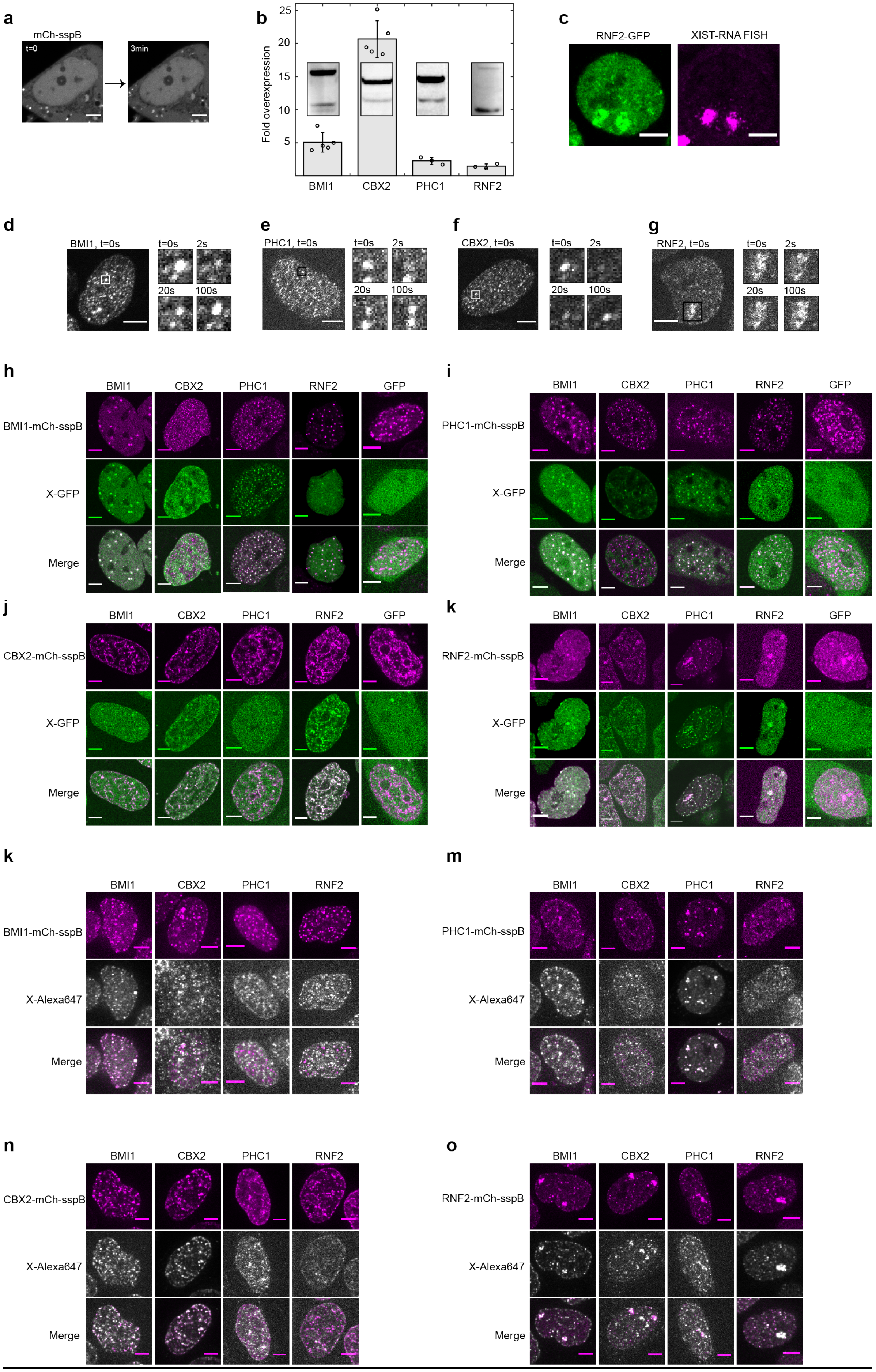
a
mCh-sspB
t=0
3min
b
Fold overexpression
25
20
15
10
5
BMI1
CBX2
PHC1
RNF2
c
RNF2-GFP
XIST-RNA FISH
d
BMI1, t=0s
t=0s
2s
20s
100s
e
PHC1, t=0s
f
CBX2, t=0s
g
RNF2, t=0s
h
BMI1-mCh-sspB
X-GFP
Merge
GFP
i
PHC1-mCh-sspB
j
CBX2-mCh-sspB
k
RNF2-mCh-sspB
k
X-Alexa647
m
n
o

Figure S1: Characterization of PRC1 corelets and colocalization analysis.

a. mCh-sspB in HEK293 cells with NLS-Ferritin-Corelet before (t=0) and after blue light activation (3min). Scale bar is 5µm.

b. Western blot quantification of overexpressed proteins. Sizes: endogenous BMI1 (43kDa), BMI1-mCh-sspB (89kDa), endogenous CBX2 (70kDa), CBX2-mCh-sspB (116 kDa), endogenous PHC1 (140kDa), PHC1-mCh-sspB (186kDa), endogenous RNF2 (41kDa), RNF2-mCh-sspB (87 kDa). (n=5/5/3/3 biological replicates for BMI1/CBX2/PHC1/RNF2). Data are presented as mean values +/- SD.

c. RNA-FISH on XIST shows that RNF2 partitions onto the inactive X-chromosomes. Scalebar is 5µm.

d. Stills of FRAP experiment BMI1-mCh-sspB. Scalebar is 5µm.

e. Stills of FRAP experiment PHC1-mCh-sspB. Scalebar is 5µm.

f. Stills of FRAP experiment CBX2-mCh-sspB. Scalebar is 5µm.

g. Stills of FRAP experiment RNF2-mCh-sspB. Scalebar is 5µm.

h. Recruitment of PRC1 subunit GFP-fusions to BMI1-mCh-sspB Corelets. Cells were activated for 3 minutes. Scalebar is 5µm.

i. Recruitment of PRC1 subunit GFP-fusions to PHC1-mCh-sspB Corelets. Scalebar is 5µm.

j. Recruitment of PRC1 subunit GFP-fusions to CBX2-mCh-sspB Corelets. Scalebar is 5µm.

k. Recruitment of PRC1 subunit GFP-fusions to RNF2-mCh-sspB Corelets. Scalebar is 5µm.

l: Recruitment of endogenous PRC1 subunits to BMI1-mCh-sspB Corelets. Scalebar is 5µm.

m: Recruitment of endogenous PRC1 subunits to PHC1-mCh-sspB Corelets. Scalebar is 5µm.

n: Recruitment of endogenous PRC1 subunits to CBX2-mCh-sspB Corelets. Scalebar is 5µm.

o: Recruitment of endogenous PRC1 subunits to RNF2-mCh-sspB Corelets. Scalebar is 5µm.

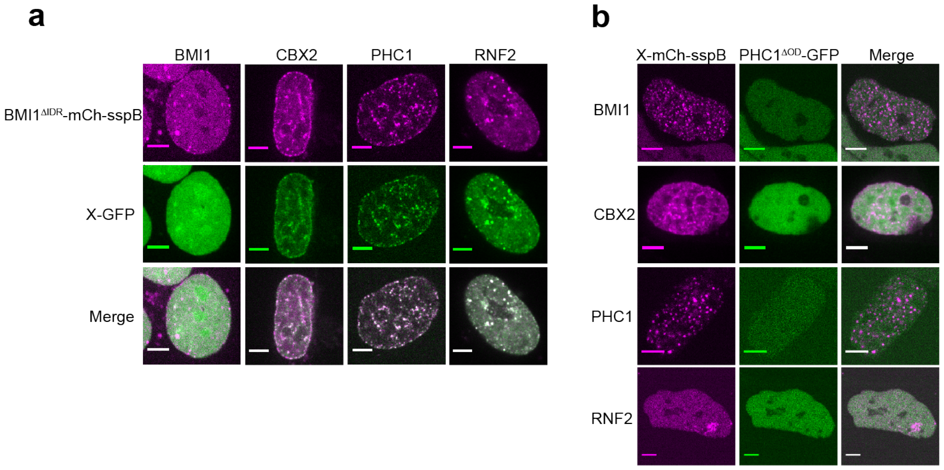

Figure S2: Colocalization of Bmi1ΔIDR and PHC1ΔOD with PRC1 proteins

a. Recruitment of PRC1 subunits to BMI1$^{\Delta IDR}$-mCh-sspB Corelets. Cells were activated for 3 minutes. Scalebar is 5µm.

b. Recruitment of PHC1$^{\Delta OD}$-GFP to PRC1-mCh-sspB Corelets. Scalebar is 5µm.

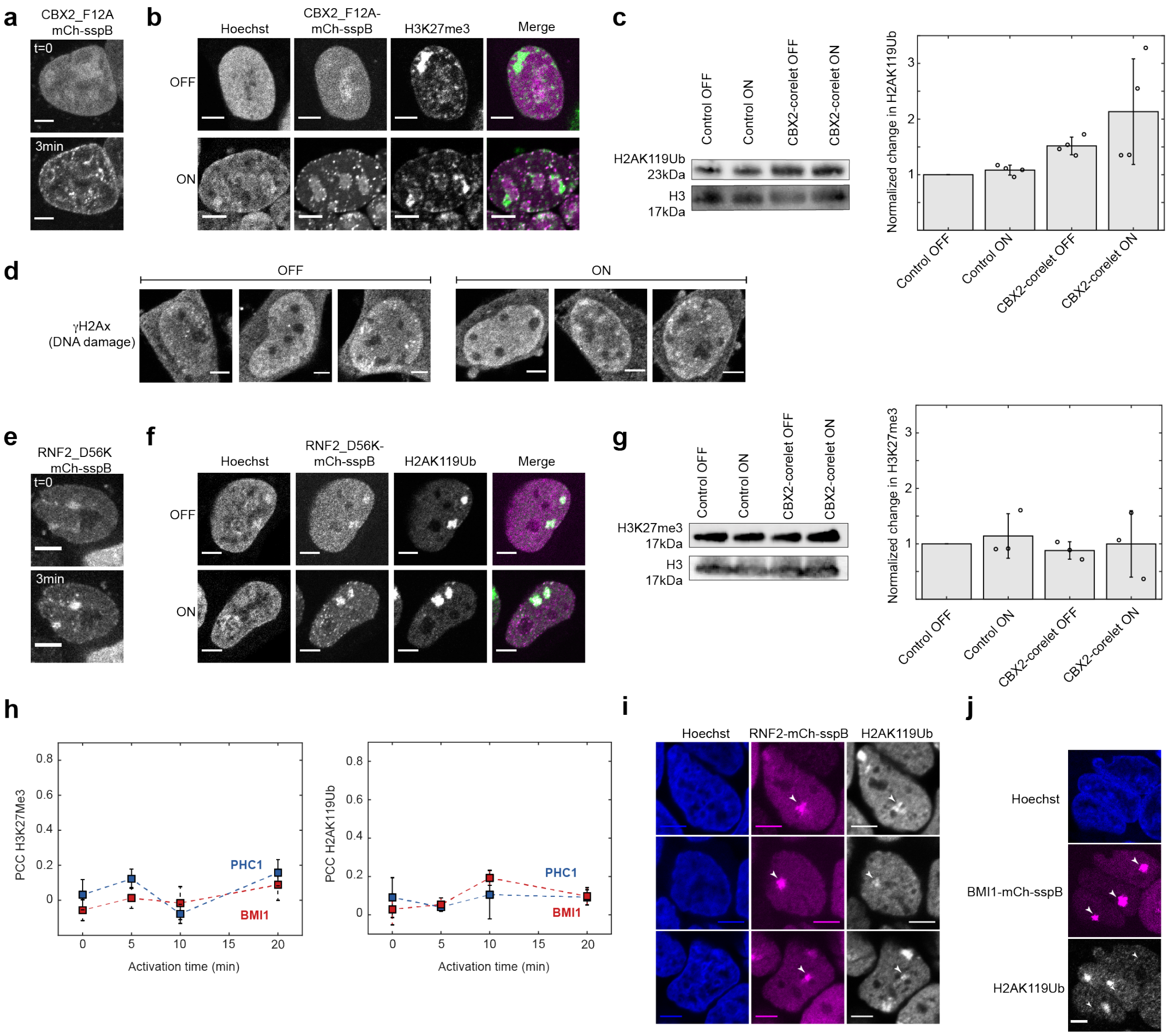

Figure S3: Colocalization of PRC1corelets with repressive histone marks.

a. CBX2^{F12A}-mCh-sspB (point mutation abolishes the ability to recognize the H3K27me3 histone mark) in HEK293 cells with NLS-Ferritin-Corelet before (t=0) and after blue light activation (3min). Scale bar is 5μm.

b. Immunofluorescence on the H3K27me3 mark in fixed CBX2^{F12A}-mCh-sspB Corelet cells, before (OFF) and after (ON) activation. CBX2^{F12A} does show increased colocalization with H3K27me3 marks. Scalebar is 5μm.

c. Western blot of control cells (GFP-corelet only) and CBX2-corelet cells, activated and non-activated. The H2AK119Ub mark increases when CBX2 is expressed and when condensates are activated. Included in the quantification are 4 biological replicates. Data are presented as mean values +/- SD.

d. Immunostain for γH2Ax, a DNA damage marker. The activation protocol does not increase DNA damage (compare OFF to ON). Scalebar is 5μm.

e. RNF2^{D56K}-mCh-sspB (point mutation that abolishes the ability to write the H2AK119Ub mark) in HEK293 cells with NLS-Ferritin-Corelet before (t=0) and after blue light activation (3 min). Scalebar is 5μm.

f. Immunofluorescence on the H2AK119Ub mark in fixed RNF2^{D56K}-mCh-sspB Corelet cells, before (OFF) and after (ON) activation. RNF2^{D56K} moderately localizes with H2AK119Ub marks. Scalebar is 5μm.

g. Western blot of control cells (GFP-corelet only) and CBX2-corelet cells, activated and non-activated. The H3K27me3 does not appear to be increasing as CBX2 is

expressed and activated. Included in the quantification are 3 biological replicates. Data are presented as mean values +/- SD.
h. Pearson-correlation coefficient of PHC1- (n=6/6/6/8 for 0/5/10/20 minutes) and BMI1-corelets (n=7/9/5/11 cells for 0/5/10/20 minutes) with H3K27me3 (left panel) and PHC1- (n=8/3/7/14 for 0/5/10/20 minutes) and BMI1-corelets (n=1011/4/18 cells for 0/5/10/20 minutes) with H2AK119Ub (right panel).
i. Three examples of the H2AK119Ub mark appearing where RNF2 is locally activated. In the bottom example, two inactive X-chromosomes are clearly distinguishable in addition to the activated spot. Scalebar is 5µm.
j. local activation of BMI1-condensates does not lead to writing of H2AK119Ub. Scalebar is 5µm.

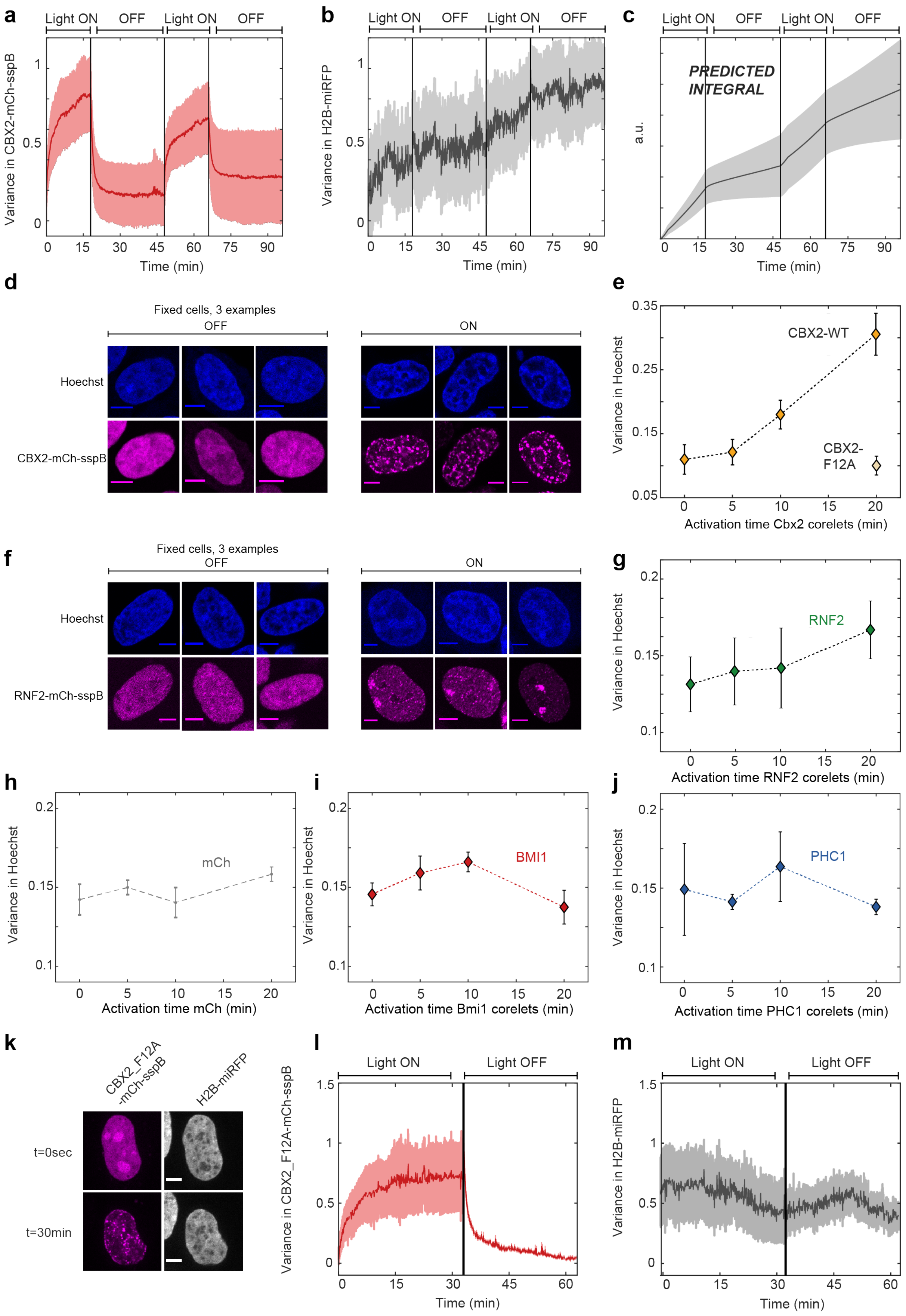

a
Light ON
OFF
Light ON
OFF
Variance in CBX2-mCh-sspB
Time (min)
b
Variance in H2B-miRFP
Time (min)
c
PREDICTED INTEGRAL
a.u.
Time (min)
d
Fixed cells, 3 examples
OFF
ON
Hoechst
CBX2-mCh-sspB
e
CBX2-WT
CBX2-F12A
Variance in Hoechst
Activation time Cbx2 corelets (min)
f
Fixed cells, 3 examples
OFF
ON
Hoechst
RNF2-mCh-sspB
g
RNF2
Variance in Hoechst
Activation time RNF2 corelets (min)
h
mCh
Variance in Hoechst
Activation time mCh (min)
i
BMI1
Variance in Hoechst
Activation time Bmi1 corelets (min)
j
PHC1
Variance in Hoechst
Activation time PHC1 corelets (min)
k
CBX2_F12A -mCh-sspB
H2B-miRFP
t=0sec
t=30min
l
Light ON
Light OFF
Variance in CBX2_F12A-mCh-sspB
Time (min)
m
Light ON
Light OFF
Variance in H2B-miRFP
Time (min)

Figure S4: Analysis of chromatin compaction after PRC1 corelet formation.
a. The variance in the CBX2-corelet signal increases as condensates form and decreases upon deactivation. Data are presented as mean +/- SD, n=29 cells.
b. The variance in the H2B-miRFP goes up as condensates form, stays relatively stable as condensates disappear, and continues on when condensates appear again. Error band represents mean +/- SD.
c. Integral of the CBX2 variance, showing that compaction sums over the prior history of CBX2 phase separation, even as compaction accumulates. Error band represents mean +/- SD.
d. Fixed CBX2-mCh-sspB Corelet cells before (OFF) and after (ON) 20 minutes of activation, stained with Hoechst. Before activation CBX2-mCh-sspB associates with homogeneously distributed chromatin throughout the nucleus. After light activation, CBX2-Corelets compact chromatin locally. Scalebar is 5μm.
e. Quantification of the variance in Hoechst increasing with CBX2-Corelet activation time (n=27/25/7/10 cells for 0/5/10/20 minutes). The CBX2-F12A mutant that is unable to interact with H3K27me3 shows compaction compared to the non-activated situation (n=18 cells). Data are presented as mean values +/- SD.
f. Before activation (OFF) the RNF2-mCh-sspB is homogeneously distributed throughout the nucleus. After light activation, RNF2-Corelets partition onto the inactive X-chromosome and form small de novo puncta throughout the nucleus. There is little change in the Hoechst distribution. Scalebar is 5μm.
g. Quantification of the variance in Hoechst increasing with RNF2-Corelet activation time (n=11/9/7/9 cells for 0/5/10/20 minutes). Data are presented as mean values +/- SD.
h. Quantification of the variance in Hoechst increasing with mCh-Corelet activation time (n=6/7/5/6 cells for 0/5/10/20 minutes). Data are presented as mean values +/- SD.
i. Quantification of the variance in Hoechst increasing with BMI1-Corelet activation time (n=7/9/8/11 cells for 0/5/10/20 minutes). Data are presented as mean values +/- SD.
j. Quantification of the variance in Hoechst increasing with PHC1-Corelet activation time (n=6/6/6/8 cells for 0/5/10/20 minutes). Data are presented as mean values +/- SD.
k. CBX2_F12A-Corelet cell with H2B-miRFP before (t=0) and after activation (30 min). Scalebar is 5μm.
l. The variance in the CBX2_F12A signal increases rapidly as condensates form, and decreases upon deactivation. Data are presented as mean +/- SD, n=6 cells.
m. The variance in the H2B-miRFP is unaffected by the condensates. Error band represents mean +/- SD.

SUPPLEMENTARY TABLE 1

List of plasmids

Plasmid	Source
FM5-NLS-iLID-mGFP-Fe	Sanders et al, Cell 2020
FM5-NLS-iLID-Fe	Sanders et al, Cell 2020
FM5-BMI1-mCh-sspB	This paper
FM5-BMI1-GFP	This paper
FM5-BMI1ΔIDR-mCh-sspB (aa 1-250)	This paper
FM5-BMI1ΔOD-mch-sspB (aa1-121+236-326)	This paper
FM5-PHC1-mCh-sspB	This paper
FM5-PHC1-GFP	This paper
FM5-PHC1ΔOD-mCh-sspB (aa 1-939)	This paper
FM5-PHC1ΔOD-GFP (aa 1-939)	This paper
FM5-CBX2-mCh-sspB	This paper
FM5-CBX2-GFP	This paper
FM5-CBX2_F12A-mCh-sspB	This paper
FM5-RNF2-mCh-sspB	This paper
FM5-RNF2-GFP	This paper
FM5-RNF2_D56K-mCh-sspB	This paper
H2B-miRFP670	Shin et al, 2018, Cell

SUPPLEMENTARY TABLE 2

List of primers

Name	Sequence
BMI1_FW	ATCACCGGTAGCTAGCACCATGCATCGAACAACGAGAATC
BMI1_REV	TACCACTACCGCTAGAACCAGAAGAAGTTGCTGA
CBX2_FW	ATCACCGGTAGCTAG**C**ACCATGGAGGAGCTGAGCAGCGTG
CBX2_REV	TACCACTACCGCTAGAGTAATGCCTCAGGTTGAA
PHC1_FW	ATCACCGGTAGCTAGCACCATGGAGACTGAGAGCGAGCAG
PHC1_REV	TACCACTACCGCTAGAGGTCTCCTTGAGGACATT
RNF2_FW	ATCACCGGTAGCTAGCACCATGACTCAGGCTGTGCAGACA
RNF2_REV	TACCACTACCGCTAGATTTGTGCTCCTTTGTAGG
BMI1dIDR_REV	TACCACTACCGCTAGATTCCAGTTCTCCAGCATT
BMI1dOD1	CTCTCTGGTGACTGATTTCATCCGCAACCTCTCC
BMI1dOD2	AGAGGTTGCGGATGAAATCAGTCACCAGAGAGAT
PHC1dOD_REV	TACCACTACCGCTGGAACGGCTAGGATTACTGGA
CBX2_F12A1	ATCACCGGTAGCTAGCACCATGGAGGAGCTGAGCAGCGTGGGCGAGC AGGTCGCCGCCGC
CBX2_F12A2	TAGTGGCCATCGATCGTGGTACCTCCTCGACTCGTCGCACCCGCTCGT CCAGCGGCGGCG
RNF2_D56K1	CCAATTTGTTTGAAGATGTTGAAGAAC
RND2_D56K2	GTTCTTCAACATCTTCAAACAAATTGG
