# Supplementary material for: Polycomb condensates can promote epigenetic marks but are not required for sustained chromatin compaction
Source: Nat Commun. 2021 Oct 7;12:5888. doi: 10.1038/s41467-021-26147-5 (PMC8497513; doi:10.1038/s41467-021-26147-5)
Supplement: Supplementary file 3 — Reporting Summary [file 41467_2021_26147_MOESM3_ESM.pdf]

nature research

Corresponding author(s): Clifford Brangwynne

Last updated by author(s): 9/3/2021

Reporting Summary

Nature Research wishes to improve the reproducibility of the work that we publish. This form provides structure for consistency and transparency in reporting. For further information on Nature Research policies, see our Editorial Policies and the Editorial Policy Checklist.

Statistics

For all statistical analyses, confirm that the following items are present in the figure legend, table legend, main text, or Methods section.

n/a	Confirmed	
☐	☒	The exact sample size (*n*) for each experimental group/condition, given as a discrete number and unit of measurement
☐	☒	A statement on whether measurements were taken from distinct samples or whether the same sample was measured repeatedly
☐	☒	The statistical test(s) used AND whether they are one- or two-sided<br>*Only common tests should be described solely by name; describe more complex techniques in the Methods section.*
☒	☐	A description of all covariates tested
☒	☐	A description of any assumptions or corrections, such as tests of normality and adjustment for multiple comparisons
☐	☒	A full description of the statistical parameters including central tendency (e.g. means) or other basic estimates (e.g. regression coefficient) AND variation (e.g. standard deviation) or associated estimates of uncertainty (e.g. confidence intervals)
☐	☒	For null hypothesis testing, the test statistic (e.g. *F*, *t*, *r*) with confidence intervals, effect sizes, degrees of freedom and *P* value noted<br>*Give P values as exact values whenever suitable.*
☒	☐	For Bayesian analysis, information on the choice of priors and Markov chain Monte Carlo settings
☒	☐	For hierarchical and complex designs, identification of the appropriate level for tests and full reporting of outcomes
☐	☒	Estimates of effect sizes (e.g. Cohen's *d*, Pearson's *r*), indicating how they were calculated

Our web collection on statistics for biologists contains articles on many of the points above.

Software and code

Policy information about availability of computer code

Data collection	Nikon elements for microscopy software, Simulations were done using the LAMMPS package (ver 3Mar20). Code is available at https://git.ecdf.ed.ac.uk/dmichiel/prc_ub_recoloring
Data analysis	All images were analysed using a combination of manual segmentation (ImageJ) and segmentation in Matlab (2017b).

For manuscripts utilizing custom algorithms or software that are central to the research but not yet described in published literature, software must be made available to editors and reviewers. We strongly encourage code deposition in a community repository (e.g. GitHub). See the Nature Research guidelines for submitting code & software for further information.

Data

Policy information about availability of data

All manuscripts must include a data availability statement. This statement should provide the following information, where applicable:

- Accession codes, unique identifiers, or web links for publicly available datasets
- A list of figures that have associated raw data
- A description of any restrictions on data availability

Source data is provided with this manuscript. Raw microscopy datasets available upon request.

Field-specific reporting

Life sciences study design

All studies must disclose on these points even when the disclosure is negative.

Sample size	Sample sizes for live cell microscopy were chosen according to commonly used and accepted standards in the field. Three independent biological replicates obtained similar results and technical replicates assured statistical robustness.
Data exclusions	Data was excluded if two independent observers disagreed on interpretation for collection of phase-diagrams.
Replication	Three independent biological replicates obtained similar results and technical replicates assured statistical robustness.
Randomization	Randomization was not feasible or necessary for this study as it involved live imaging of cells by a single investigator.
Blinding	Image analysis for phase diagram construction was repeated by a second observer who was blind to sample identity. Blinding was not feasible for other parts as they were performed by a single investigator.

Reporting for specific materials, systems and methods

We require information from authors about some types of materials, experimental systems and methods used in many studies. Here, indicate whether each material, system or method listed is relevant to your study. If you are not sure if a list item applies to your research, read the appropriate section before selecting a response.

Materials & experimental systems

n/a	Involved in the study	
☐	☒	Antibodies
☐	☒	Eukaryotic cell lines
☒	☐	Palaeontology and archaeology
☒	☐	Animals and other organisms
☒	☐	Human research participants
☒	☐	Clinical data
☒	☐	Dual use research of concern

Methods

n/a	Involved in the study	
☒	☐	ChIP-seq
☒	☐	Flow cytometry
☒	☐	MRI-based neuroimaging

Antibodies

Antibodies used	Anti-H3K27Me3, rabbit Cell signal #9733<br>Anti-H2AK119Ub, rabbit Cell signal #8240<br>Anti-Bmi1, rabbit Cell signal #6964<br>Anti-Cbx2, rabbit Sigma Aldrich HPA023083<br>Anti-PHC1, rabbit Sigma Aldrich HPA006973<br>Anti-RNF2, rabbit Cell signal #5694<br>Anti-Rabbit IgG, Alexa fluor 647goat Thermo Fisher A-21245<br>Anti-H3, rabbit Cell signal #4499<br>Peroxidase-AffiniPure Goat Anti-Rabbit IgG Jackson ImmunoResearch Europe Ltd. 111-035-144
Validation	Validation of the antibodies used in this study was done by the manufacturer and the details of these validation efforts are described on the manufacturer's website

Eukaryotic cell lines

Policy information about cell lines

Cell line source(s)	ATCC: HEK293, HEK293T
Authentication	Seventeen short tandem repeat (STR) loci plus the gender determining locus, Amelogenin, were amplified using the commercially available PowerPlex® 18D Kit from Promega. The cell line sample was processed using the ABI Prism® 3500xl Genetic Analyzer. Data were analyzed using GeneMapper® ID-X v1.2 software (Applied Biosystems). Appropriate positive and negative controls were run and confirmed for each sample submitted
Mycoplasma contamination	Cells were tested negative for mycoplasma
Commonly misidentified lines (See ICLAC register)	NA
